# Single-Dose St. Thomas Versus Custodiol® Cardioplegia for Right Mini-thoracotomy Mitral Valve Surgery

**DOI:** 10.1007/s12265-022-10296-z

**Published:** 2022-08-08

**Authors:** Cristina Barbero, Marco Pocar, Giovanni Marchetto, Erik Cura Stura, Claudia Calia, Bianca Dalbesio, Claudia Filippini, Stefano Salizzoni, Massimo Boffini, Mauro Rinaldi, Davide Ricci

**Affiliations:** 1 Cardiovascular and Thoracic Department, Division of Cardiac Surgery, , Città Della Salute E Della Scienza”, University of Turin, Corso Dogliotti, 14 Turin, Italy; 2 Department of Surgical Sciences, University of Turin, Turin, Italy; 3 Department of Clinical Sciences and Community Health, University of Milan, Milan, Italy; 4 Department of Integrated Surgical and Diagnostic Sciences, University of Genova, Genoa, Italy

**Keywords:** Minimally invasive surgery, Crystalloid cardioplegia, Myocardial protection, Cardiopulmonary bypass, Mitral valve repair

## Abstract

**Objective:**

Custodiol® and St. Thomas cardioplegia are widely employed in mini-thoracotomy mitral valve (MV) operations. One-dose of the former provides 3 h of myocardial protection. Conversely, St. Thomas solution is usually reinfused every 30 min and safety of single delivery is unknown. We aimed to compare single-shot St. Thomas versus Custodiol® cardioplegia.

**Methods:**

Primary endpoint of the prospective observational study was cardiac troponin T level at different post-operative time-points. Propensity-weighted treatment served to adjust for confounding factors.

**Results:**

Thirty-nine patients receiving St. Thomas were compared with 25 patients receiving Custodiol® cardioplegia; cross-clamping always exceeded 45 min. No differences were found in postoperative markers of myocardial injury. Ventricular fibrillation at the resumption of electric activity was more frequent following Custodiol® cardioplegia (*P* = .01).

**Conclusion:**

Effective myocardial protection exceeding 1 h of ischemic arrest can be achieved with a single-dose St. Thomas cardioplegia in selected patients undergoing right mini-thoracotomy MV surgery.

## Introduction

Adequate and safe myocardial protection is essential to achieve optimal results in cardiac surgery, regardless of the type of surgical procedure and the approach. Suboptimal myocardial protection during cardiopulmonary bypass is an independent predictor of perioperative adverse events, including myocardial infarction, ischemia–reperfusion injury, arrhythmias, and low cardiac output syndrome, which are associated with prolonged intensive care unit and hospital length of stay, and increased mortality [1].

With respect to right mini-thoracotomy mitral valve (MV) surgery, the most adequate cardioplegic solution and delivery route have been widely debated. In the minimally invasive setting, the possibility to employ a single-dose cardioplegia is definitely appealing, allowing an uninterrupted operation and avoiding cumbersome and time-consuming repositioning of the atrial retractor to ensure proper delivery of the solution, and hazardous displacement of the endo-aortic clamp (EAC), when used.

Both Custodiol® and St. Thomas 1 crystalloid cardioplegic solutions are currently used at our Institution in right mini-thoracotomy cardiac operations. A single dose of Custodiol® enables up to 3 h of safe cardioplegic arrest and it is consequently chosen in case prolonged aortic cross-clamping is anticipated, most commonly in case of complex MV repair [2–9]. However, the overall safety and efficacy of Custodiol® compared to St. Thomas and cold blood cardioplegia remains debated, and controversial data have been reported in animals and humans [10–12]. Moreover, concerns have been raised regarding hyponatremia and acidosis following the rapid administration of the required high volume of the Custodiol® solution [9, 13]. Conversely, main drawback of the St. Thomas solution is the need for repetitive dosing (every 30 min) to ensure adequate myocardial protection. In the case cardioplegic arrest is judged to last less than 60 min, however, single-shot St. Thomas solution administration has been largely employed in right mini-thoracotomy cardiac operations at our Institution during the past 15 years. Interestingly, effective myocardial protection has been observed also after longer than anticipated cross-clamp times.

The aim of this study was to compare the efficacy and safety of myocardial protection provided by a single-dose administration of St. Thomas compared to Custodiol® solution in patients undergoing right mini-thoracotomy MV surgery.

## Materials and Methods

### Study Design

A single-institution, prospective observational study was performed on patients undergoing right mini-thoracotomy MV surgery and myocardial protection with a single dose of St. Thomas 1 or Custodiol® cardioplegic solutions. Patient enrollment was undertaken during a 5-year span between 2014 and 2018.

Primary outcome was the evaluation of cardiac troponin T (cTn-T) levels measured at different time-points after surgery, i.e., immediately after weaning from cardiopulmonary bypass and 6, 12, and 24 h thereafter. Secondary outcomes were ventricular fibrillation as first rhythm after cross clamp release, the occurrence of post-operative myocardial infarction, postoperative creatinine kinase-myocardial band (CK-MB), creatinine kinase (CK), and lactate levels at the same time-points, the occurrence of low cardiac output syndrome, and the need for postoperative inotropic support.

Patients undergoing MV surgery via right mini-thoracotomy were included in the study. Exclusion criteria were urgent or emergency surgery, left ventricular ejection fraction lower than 40%, more than mild aortic regurgitation, any history of coronary artery disease, and concomitant atrial fibrillation ablation. Associated procedures included tricuspid surgery and/or atrial septal defect closure. Reoperations were included except for prior coronary artery bypass grafting, irrespective of patent grafts. Patients requiring a second cross-clamping or who received a repeated administration of St. Thomas 1 cardioplegia for longer than expected ischemic times were also dropped out. The study protocol was reviewed and approved by the Institutional Ethics Committee (protocol number 0063123, June 18, 2014).

All patients were assessed with a preoperative electrocardiogram (ECG) and echocardiogram. After the surgical procedure, patients were investigated for ECG abnormalities and new-onset hypokinetic or akinetic segments at echocardiography.

During the study period, minimally invasive mitral valve surgery was performed by 3 surgeons; the study was designed at the end of the learning curve of all of them.

### Definitions

Postoperative myocardial infarction was defined as cTn-T value > 10 times the 99^th^ percentile of the upper reference limit during the first 48 h with ECG abnormalities and/or angiographic or imaging evidence of new myocardial ischemia/new loss of myocardial viability [14]. Upper reference limit for CK-MB and for cTn-T were five ng/mL and 50 ng/L, respectively. Low cardiac output syndrome refers to cardiac index lower than two L/min/m^2^ and systolic blood pressure lower than 90 mmHg, in conjunction with signs of tissue hypoperfusion (cold periphery, clammy skin, confusion, oliguria, elevated lactates level) in the absence of hypovolemia [15]. Inotropic support was administered with the primary goal of maintaining a mean arterial pressure above 60 mmHg and cardiac index more than two L/min/m^2^. Vasoactive-inotropic score indicates the amount of cardiovascular support by various inotropes or vasopressors during the first 24 h after the surgical procedure. The score was calculated as follows: dopamine dose (μg/kg/min) + dobutamine dose (μg/kg/min) + 100 × epinephrine dose (μg/kg/min) + 10 × milrinone dose (μg/kg/min) + 10.000 × vasopressin dose (U/kg/min) + 100 × norepinephrine dose (μg/kg/min) [16]. Postoperative stroke refers to a new, permanent neurological disability or deficit. Perioperative mortality included all deaths occurring during hospitalization or within 30 days of the procedure.

### Surgical Procedure and Myocardial Protection

Right mini-thoracotomy approach with perfusion strategies and aortic clamping techniques used at our Institution have been previously described [17–19]. All comers for MV surgery routinely undergo a full preoperative work-up for the minimally invasive approach based upon clinical history and anatomy with the aim to be allocated either to retrograde arterial perfusion with transthoracic or EAC or antegrade arterial perfusion with transthoracic aortic cross-clamping [17–19].

The choice of Custodiol® (25 ml/kg) or St. Thomas 1 (1 L induction dose, 500 ml maintenance when repeated) cardioplegia for myocardial protection was dictated by the estimated complexity of the procedure, and thus of ischemic time, and on the surgeon’s preference. Custodiol® is primarily preferred in case of planned prolonged procedures, typically complex MV repair, because a single dose may enable up to 3 h of safe cardiac arrest before redosing; St. Thomas is usually preferred in case of planned less than 60-min procedures, such as MV replacement or simple MV repair.

Both cardioplegic solutions were administered as a single dose, with no additional topical cooling, nor retrograde cardioplegia through the coronary sinus in any patient. In the Custodiol® group, hyponatremia was treated with a hemofilter on the cardiopulmonary bypass circuit, or prevented by direct aspiration of cardioplegia through a minimal right atrial incision and temporary bicaval snaring. All patients were systemically cooled to 30 °C. Clamp release was obtained at the end of appropriate air venting at a body temperature above 33 °C. All patients received 20 g of mannitol intravenously, after starting cardiopulmonary bypass and during rewarming, after reaching 33 °C or higher core temperatures.

### Statistical Analysis

Continuous data are presented as mean and standard deviation or median and interquartile range depending on data distribution, whereas categorical variables are presented as rate and proportion. In univariate analysis continuous variables were compared using unpaired *t* or Wilcoxon-Mann–Whitney tests according to distribution type. Categorical variables were compared with the use of the chi-square test or Fisher exact test, as appropriate.

A matched analysis using propensity score was performed to reduce possible differences between groups and obtain unbiased estimation of the treatment effect. The propensity score of receiving St. Thomas versus Custodiol® solution was estimated using multivariable logistic regression analysis with the type of solution as dependent variable. The a priori selected variables were as follows: age, gender, vasculopathy, Euroscore II, previous cardiac surgery procedures, ejection fraction, subtype of MV disease, and technique of aortic clamping. All variables were obtained before right mini-thoracotomy MV surgery. The genetic matching method without replacement was used to match patients treated with St. Thomas and Custodiol® solution [20]. Genetic matching method achieves covariates balance and minimizes bias related to data replacement or arbitrary caliper matching [21]. Analyses were performed before and after matching. Standard differences were used to test balance and to compare the difference in the covariate between the pre- and post-matched sample, since the calculation is not dependent on sample size. A standardized difference is the difference in means between groups, dived by the (pooled) standard deviation: values < 0.1 indicate adequate balance [22].

To examine the temporal effect across groups, St. Thomas solution versus Custodiol® solution during the 24-h observation period after surgery, a mixed-linear regression model for repeated measures was applied. All statistical tests were two-sided and *p* values of 0.05 or less were considered statistically significant. Stata (Stata-Corp, College Station, TX), R Package “MatchIt” (R Foundation for Statistical Computing, http://www.r-project.org/), and SAS (SAS Institute, Cary, NC) software packages were used for computations.

## Results

**Fig. 1:**
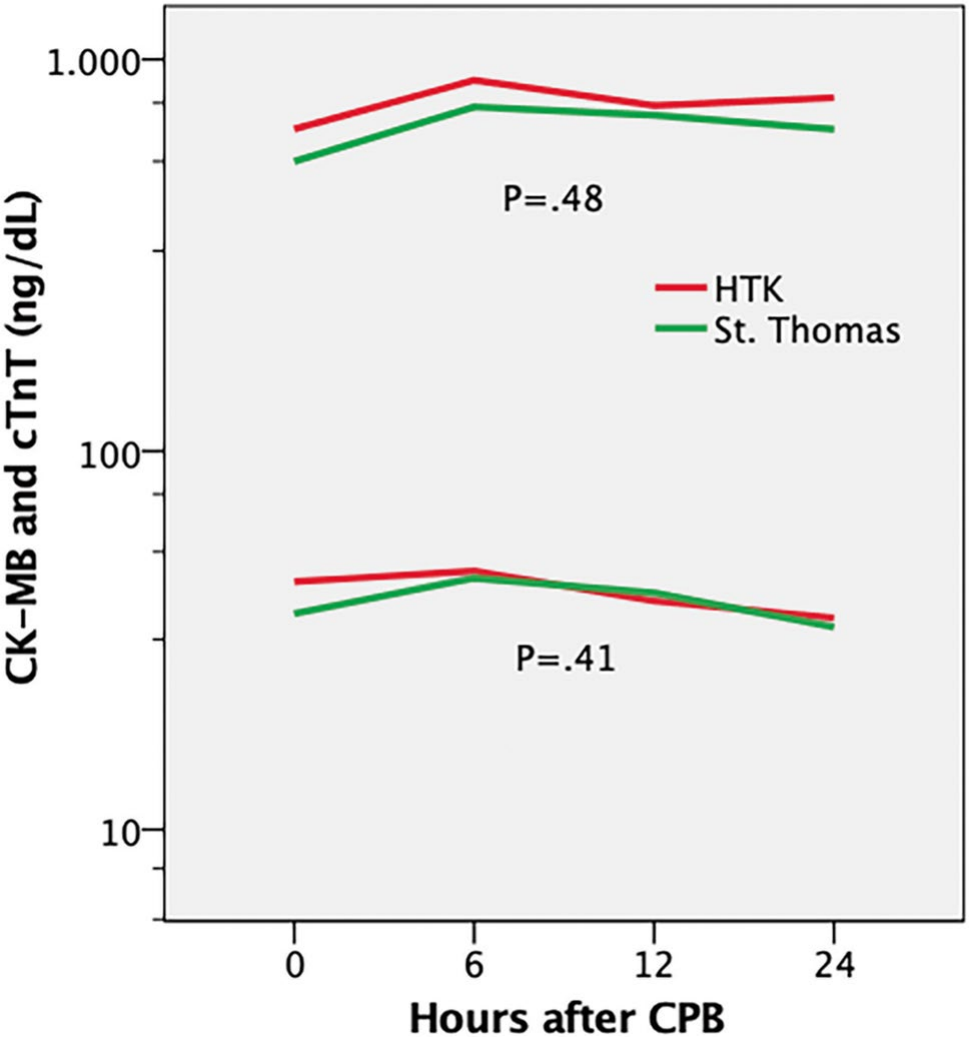
cTn-T and CK-MB activities. cTn-T: cardiac Troponin T; CK-MB: creatinine kinase-myocardial band; HTK: hystidine-triptophan-ketoglutarate

During the study period, 566 patients underwent right mini-thoracotomy MV surgery at our Institution. One-hundred eighty-seven patients met the inclusion criteria for the study, with 39 and 148 patients receiving St. Thomas 1 or Custodiol® solution, respectively. Before matching, groups were not comparable with respect to age, gender, baseline creatinine, peripheral vascular disease, atrial fibrillation, EuroSCORE II, previous cardiac surgery and ejection fraction (Table 1). After matching, all 39 patients in the St. Thomas group were compared with 25 patients receiving Custodiol® cardioplegia. Treatment groups were similar with respect to preoperative characteristics, type of MV surgery and combined procedures, aortic clamping technique, and cardiopulmonary bypass and aortic cross-clamp times (Tables 1 and 2). No cases of myocardial infarction or low cardiac output syndrome were reported. No ECG abnormalities or new hypokinetic or akinetic area were highlighted on postoperative echocardiograms. No differences between groups were recorded in terms of inotropic support requirements, intensive care unit or hospital length of stay, and perioperative mortality, whereas a higher incidence of spontaneous ventricular fibrillation after aortic clamp release was observed in the Custodiol® group in spite of a mean cross-clamp time of 84 ± 19 min (5 cases versus none, *p* = 0.01). Aortic cross-clamp time exceeded 45 min in all patients, and was less than 60 min in only eight. Median cross-clamp times were 73 min (*Q*_1_–*Q*_3_, 60–90) in the St. Thomas group and 80 min (*Q*_1_–*Q*_3_, 64–99) in the Custodiol® group. Finally, no differences were found in the release of myocardial markers, lactate levels, and vasoactive-inotropic score at different time-points after surgery (Table 3 and Fig. 1).

**Table 1:** Patients’ characteristics before and after matching

Characteristic		Before matching		After matching	
	St. Thomas	HTK	*St diff*	HTK	*St diff*
	(*n* = 39)	(*n* = 148)		(*n* = 25)	
Age (yrs)	68.6 ± 13.4	61.4 ± 12.4	0.56	65.8 ± 15.3	0.19
Female	24 (61.5%)	51 (34.5%)	0.56	14 (56%)	0.11
EuroSCORE II (%)	9.7 ± 11.3	3.9 ± 3.1	0.71	6.6 ± 4.1	0.38
Ejection fraction (%)	58.5 ± 8.4	62.9 ± 7.1	- 0.58	61.8 ± 4.5	- 0.50
Previous cardiac surgery	6 (15.4%)	4 (2.7%)	0.45	1 (4%)	0.39
Peripheral vascular disease	14 (35.9%)	30 (20.3%)	0.35	10 (40%)	- 0.08
Endo-aortic clamp	12 (30.8%)	79 (53.4%)	- 0.33	7 (28%)	- 0.06
Aortic cross-clamp time (min)	78.1 ± 24.5	100.5 ± 25.6	- 0.90	84.2 ± 23.6	- 0.25

*HTK*, hystidine-triptophan-ketoglutarate; *St diff*, standardized mean difference

**Table 2:** Baseline characteristics, surgical data, and outcome after propensity score matching

Characteristic	St. Thomas	HTK	*P* value
	(*n* = 39)	(*n* = 25)	
BMI (kg/m2)	25.2 ± 3.4	24.4 ± 4.3	.48
Creatinine (mg/dL)	1.1 ± 0.3	1 ± 0.5	.45
CKD ≥ 4 or dialysis	5 (12.8%)	2 (8%)	.69
Diabetes	6 (15.4%)	5 (20%)	.79
COPD	3 (7.7%)	3 (12%)	.67
Mitral valve repair	11 (47.8%)	11 (44%)	.28
Tricuspid valve surgery	4 (10.3%)	4 (16%)	.70
ASD closure	4 (10.3%)	1 (4.2%)	.64
CPB time (min)	103 (88–128)	116 (102–139)	.11
Cross-clamp time (min)	73 (60–90)	80 (64–99)	.27
Endo-aortic clamp	12 (30.8%)	7 (28%)	> .99
Fibrillation after unclamping	0	5 (20%)	.01
Mechanical ventilation (hrs)	12 (10–17)	11 (10–16)	.51
Mechanical ventilation > 72 h	6 (15.4%)	0	.07
Inotropic support	31 (79.5%)	20 (80%)	.61
Stroke	1 (2.6%)	0	> .99
Dialysis	2 (5.1%)	1 (4.2%)	> .99
Re-exploration for bleeding	1 (2.6%)	1 (4.2%)	> .99
ICU stay (days)	1 (1–2)	1 (1–1)	.15
Hospital stay (days)	7 (6–8)	7 (6–8)	.93
Perioperative mortality	1 (2.6)	0	> .99

*HTK*, hystidine-triptophan-ketoglutarate; *BMI*, body mass index; *CKD*, chronic kidney disease; *COPD*, chronic obstructive pulmonary disease; *ASD*, atrial septal defect; *CPB*, cardiopulmonary bypass; *ICU*, intensive care unit

**Table 3:** Markers of myocardial injury and cardiac output at different postoperative time-points

	Hours after CPB				*P* value
	0	6	12	24	
cTn-T (ng/mL)					
St. Thomas	550.6 (496)	757.4 (603.3)	721 (564.2)	664.6 (651.2)	
HTK	665.9 (380.3)	885.9 (436.6)	763.2 (483.2)	799.5 (834)	.48
CK-MB (ng/mL)					
St. Thomas	38.4 (30.5)	46.9 (36)	43.4 (32)	34.6 (28.6)	
HTK	45.8 (28.2)	49.6 (24.7)	41.1 (19.3)	36.7 (20.4)	.41
CK (U/L)					
St. Thomas	986.5 (595.2)	1135.1 (669.5)	1107.3 (617.2)	892.9 (619.5)	
HTK	1325.5 (633.6)	1362 (710.1)	1243.1 (859.7)	1069.5 (806.6)	.10
VIS					
St. Thomas	7.9 (14.3)	9.1 (14.2)	9.1 (16.9)	7.8 (18.4)	
HTK	8.32 (10.9)	9.5 (14.5)	5.3 (10.4)	1.7 (3.3)	.53
Lactate (mmol/L)					
St. Thomas	1.6 (1.5)	1.6 (1.8)	2 (1.7)	1.8 (1.5)	
HTK	1.1 (0.8)	1.4 (0.7)	1.8 (1.3)	1.4 (0.9)	.30

*CPB*, cardiopulmonary bypass; *cTn-T*, cardiac troponin T; *HTK*, hystidine-triptophan-ketoglutarate; *CK-MB*, creatine kinase myocardial band; *CK*, creatine kinase; *VIS*, vasoactive-inotropic score

Data are expressed as mean and standard difference

## Discussion

Our approach for right mini-thoracotomy cardiac surgery has been standardized over the last 15 years in more than 1500 cases with the use of St. Thomas type 1 or Custodiol® crystalloid solutions for myocardial protection. The type of cardioplegia is chosen according to the anticipated duration of the procedure and to the surgeon’s preference. In comparison with other cardioplegic strategies, St. Thomas or Custodiol® cardioplegia may allow the avoidance of multiple doses administration, which intrinsically necessitates repetitive repositioning of the heart and left atrial retractor for adequate cardioplegia delivery. These maneuvers are particularly tedious and time-consuming in the minimally invasive setting, and may ultimately favor the deleterious consequences of prolonged myocardial ischemia. Moreover, they can cause dislodgment of the EAC, when used, increasing the risk of myocardial suboptimal perfusion.

Several studies comparing different types of cardioplegia have been reported with controversial results [23–25]. A recent multivariate analysis from the Society of Thoracic Surgeons (STS) database on 4976 isolated minimally invasive MV procedures showed that crystalloid and blood/crystalloid cardioplegia were associated with significant increased risk of mortality and of longer ICU stay compared with blood cardioplegia [1]. However, myocardial biomarkers and postoperative cardiac function are lacking in the STS database, and the specific type of solution administered for cardiac protection is not precisely reported. For example, the Del Nido solution is categorized as “blood cardioplegia”, despite a blood/crystalloid ratio of 1:4 [26]. These biases may potentially weaken the results of the analysis.

On the counterpart, other studies comparing crystalloid and cold blood cardioplegic solutions in patients undergoing right mini-thoracotomy MV operations highlighted equivalent or even better outcomes in the former in terms of mortality, myocardial injury enzyme release, arrhythmias, and early complications [7, 23, 27–30]. These issues are crucial in minimally invasive surgery, particularly with the EAC setting, where a blood solution may significantly impact the delivery line resistances, and repeated administration of cardioplegia could lead to displace the EAC itself causing suboptimal coronary arteries perfusion.

In the minimally invasive setting, a single-shot, long-lasting dose of crystalloid cardioplegia is definitely straightforward and less time-consuming; therefore, crystalloid solutions are largely preferred. Custodiol® is primarily preferred in case of planned prolonged procedures, typically complex MV repair, because a single dose may enable up to 3 h of safe cardiac arrest before redosing. However, concerns have been raised regarding the adequate myocardial protection of the Custodiol® cardioplegia when compared to other crystalloid solutions [23, 31]. On the other hand, the administration of the St. Thomas cardioplegia is generally considered safe if repeated every 30 min [32].

Only few data exist in the literature regarding a direct comparison between Custodiol® and St. Thomas cardioplegia. In a recent study focused on minimally invasive MV operations, the St. Thomas solution was associated with lower postoperative cTn-T peak levels [31]. Interestingly, the authors employed the St. Thomas 2 solution as a single shot, advocating effective and safe myocardial protection up to 2 h of cardioplegic arrest. Similarly, we administered both solutions as a single dose and cross-clamp times exceeded 45 min in all patients after matching the groups. Besides, median aortic cross-clamp times were 80 min in the Custodiol® group and 73 min in the St. Thomas group, respectively. After propensity-weighted adjustment, we observed no differences in myocardial markers at specific time-points after surgery, lactate levels, inotropic support, and early postoperative complications. Although all cardiac markers were higher in the Custodiol® group, the difference did not reach significance.

In contrast, others have reported superior results with Custodiol® compared to repeated-dose St. Thomas 2 cardioplegia [33, 34]. In both comparative studies, however, the patient population was substantially different because it referred to isolated coronary artery bypass grafting in one case and complex congenital heart surgery in pediatric patients in the other. In the latter, moreover, systemic cooling was carried out reaching lower temperatures of 18–20 °C and mean cross-clamp times exceeded two-and-a-half hours and were always longer than 2 h. Consequently, results are unlikely to be compared with minimally invasive MV valve operations in patients with healthy vasculature and no coronary lesions. 

The only significant difference recorded was a higher rate of ventricular fibrillation as first rhythm after aortic clamp release in the Custodiol® group, in analogy to previous reports which demonstrated how the Custodiol® solution may trigger ventricular fibrillation during myocardial reperfusion even when not associated with high postoperative troponin levels [6, 8, 24].

Speculatively, the procaine contained in the St. Thomas 1 solution might have a protective role in this respect, especially if the aorta is unclamped during rewarming in near-normothermic conditions.

St. Thomas solution administrated in one dose should be prefer in cases in which the ischemic time is planned to be shorter than 1 h; it allows the surgeon to maintain the benefits of a single dose crystalloid cardioplegia and allows to avoid the risk of hyponatremia and acidosis related to the Custodiol® solution, and to avoid the need for caval snaring and right atrium incision for the drainage of the solution. Our experience shows that both type of cardioplegia should be part of an effective program of minimally invasive MV surgery and the choice of the type of cardioplegia to deliver should be adapt on the surgical procedure.

Main limitations of this study are the single-center observational design and the relatively small sample size. Moreover, no data regarding more sensitive tools to asses left ventricular function, such as echocardiography-based speckle-tracking strain imaging, are reported.

## Conclusions

Custodiol® and St. Thomas cardioplegic solutions are largely employed for myocardial protection during minimally invasive cardiac surgery: both allow surgeons to minimize the need for repeated dosing maintaining optimal exposure of the surgical field throughout the procedure and are congruent with the EAC setting where a blood cardioplegic solution may significantly impact the delivery line resistances and the balloon positioning. Effective myocardial protection exceeding 1 h of ischemic arrest can be achieved with a single-dose St. Thomas 1 solution. Results are comparable to Custodiol® solution in selected patients undergoing right mini-thoracotomy MV surgery.

## Abbreviations

MV: Mitral valve
EAC: Endo-aortic clamp
cTn-T: Cardiac troponin T cTn-T
CK-MB: Creatinine kinase-myocardial band
ECG: Electrocardiogram
